# A novel approach to prepare Bi_2_Fe_4_O_9_ flower-like spheres with enhanced photocatalytic performance

**DOI:** 10.1038/s41598-017-00831-3

**Published:** 2017-04-10

**Authors:** Haibo Yang, Jingjing Dai, Lei Wang, Ying Lin, Fen Wang, Pan Kang

**Affiliations:** School of Materials Science and Engineering, Shaanxi University of Science and Technology, Xi’an, 710021 PR China

## Abstract

A novel two-step approach consisting of hydrothermal process and subsequently selective etching has been developed to prepare flower-like three-dimensional porous Bi_2_Fe_4_O_9_ spheres with good uniformity and highly photocatalytic performance. XRD patterns and SEM images reveal that the Bi_2_Fe_4_O_9_ phase does not exhibit any changes after the etching process, and the crystal morphology evolves from micro-platelets to flower-like three-dimensional porous Bi_2_Fe_4_O_9_ spheres by controlling the experiment parameters. The change of morphology will lead to the significant increase of specific surface area, which would be beneficial to the enhancement of photocatalytic performance owing to prominent absorption in the ultraviolet and visible light region. As compared to Bi_2_Fe_4_O_9_ microplatelets, flower-like three-dimensional porous Bi_2_Fe_4_O_9_ spheres exhibit excellent photocatalytic degration rate of methyl orange (MO).

## Introduction

For the sustainable development of human society, the development of both pollution-free technologies for environmental remediation^1^ and alternative clean energy supplies is an urgent task. Moreover, visible-light-driven photocatalysts have been developed for efficient utilization of solar energy to address the increasing environmental pollution and energy problems. Among the extensive variety of green earth and renewable energy projects underway, semiconductor photocatalysis^2–4^ has emerged as one of the most promising technologies because it represents an easy way to utilize the energy of either natural sunlight or artificial indoor illumination, and is thus abundantly available everywhere in the world^5–9^.

It is believed that the properties of functional materials strongly depend on their morphology, microstructure, dimension, crystallinity, and so forth^10–15^. The ability to control particle morphology is an important objective in particle synthesis, since size and shape can significantly influence photocatalytic properties^16–18^. Liu *et al*.^19^ fabricated a magnetically separable photocatalyst based on nest-like **γ**-Fe_3_O_4_/ZnO double-shelled hollow structures with enhanced photocatalytic activity. Yu *et al*.^20^ prepared TiO_2_ solid spheres and hollow microspheres by typical hydrothermal processes in NH_4_F aqueous solution and investigated their visible-light-driven photocatalytic activities. It turns out that the photocatalytic activity of the samples prepared in the presence of NH_4_F is greatly higher than that of TiO_2_ sample prepared in pure water and commercial Degussa P25 (P25) powders. Porous cerium dioxide hollow spheres based on the Ostwald ripening process were fabricated by a simple solvothermal method in the absence of any templates. As compared to cerium dioxide nanoparticles, porous cerium dioxide hollow spheres have an enhancement for the light harvesting and provide activity sites in the photocatalytic process^21^. BiFeO_3_ nanoparticles ranging from 80 to 120 nm synthesized via a simple sol-gel method by Gao *et al*.^22^. It were demonstrated that its degradation was significantly more efficient than that of bulk BiFeO_3_ due to the higher surface area of nanosized BiFeO_3_. Besides, Reitz *et al*.^23^ studied the effect of porosity on the photocatalytic degradation of a common dye. In addition, some others previous studies also focused on the relationship between morphology and photocatalytic properties^24–27^. According to the above-mentioned studies, the enhancement of photocatalytic properties can be achieved by controlling the morphology of photocatalyst.

Bi_2_Fe_4_O_9_, as a typical semiconductor, is well-known for its catalytic performance for ammonia oxidation to NO^28–30^. It has orthorhombic structure with a space group of Pbam, which consists of two formula units per unit cell. The formula units can be described as columns of edge sharing FeO_6_ octahedra connected by corner sharing FeO_4_ tetrahedra and bismuth ions and the bismuth ions are surrounded by eight oxygen ions with mutually orthogonal shorter BiO_3_ and longer BiO_5_ units^13, 31^. In addition, Bi_2_Fe_4_O_9_ possesses the ability to photodegrade aqueous ammonia and MO under the ultraviolet light (UV-light) because of its relatively small bandgap for absorption of light^13, 32^. However, the photocatalytic degradation efficiency of the Bi_2_Fe_4_O_9_ with the regular shape is not high due to a prompt recombination of h^+^ and e^−^ generated from light^33–37^.

In this work, the flower-like three-dimensional porous Bi_2_Fe_4_O_9_ spheres have been synthesized simply by etching Bi_2_Fe_4_O_9_ microplatelets. As compared to the unetched counterparts, the flower-like three-dimensional porous Bi_2_Fe_4_O_9_ spheres exhibit excellent photocatalytic degration rate of MO under ultraviolet and visible light irradiation.

## Results and Discussion

The formation process of the three-dimensional flower-like Bi_2_Fe_4_O_9_ spheres was investigated by time-dependent evolution experiments. Intermediate products were collected at different stages, and their phase, morphology and structure informations were subjected to the following sections.

**Figure 1:**
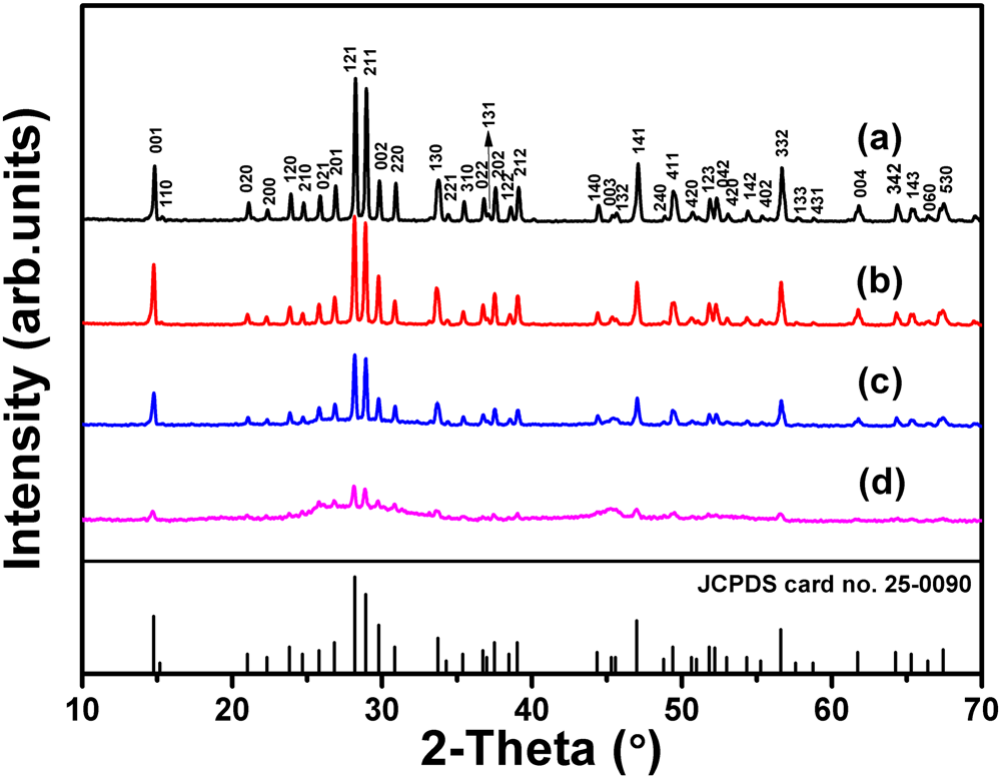
XRD patterns of the obtained Bi_2_Fe_4_O_9_ samples with different etching times: (**a**) 0; (**b**) 15 min; (**c**) 30 min; (**d**) 45 min.

The phase purity of the prepared samples with different etching times was estimated by the XRD patterns (Fig. 1). As can be seen from the patterns, all the diffraction peaks can be perfectly indexed to the orthorhombic (space group: Pbam) structure of bulk Bi_2_Fe_4_O_9_ with lattice contants of a = 7.965 Å, b = 8.44 Å, c = 5.994 Å, which is consistent with the standard data (JSPDS 25-0090). As displayed in Fig. 1, the sharp peaks in the XRD patterns indicate that the bulk Bi_2_Fe_4_O_9_ powders prepared by the hydrothermal method are well-cystallized and have no any impurities. It can be also seen that the etched Bi_2_Fe_4_O_9_ still maintain the pure phase, but the intensity of characteristic peaks shows an obvious decrease with increasing the etching time. It may be ascribed to the porous structure of the etched Bi_2_Fe_4_O_9_ spheres, which contain nano-sized fragments and tiny miscrospheres.

The phase of the samples with the different etching times is also proved by Raman spectra (Figure S1, Supplementary Information). According to the group theory, the orthorhombic Bi_2_Fe_4_O_9_ possess 42 Raman active modes (12A_g_ + 12B_1g_ + 9B_2g_ + 9B_3g_)^38, 39^. As shown in Figure S1a, there are seven modes located at 202 cm^−1^, 279 cm^−1^, 318 cm^−1^, 357 cm^−1^, 416 cm^−1^, 537 cm^−1^, 643 cm^−1^ in bulk Bi_2_Fe_4_O_9_ phase. As etching time goes on, it can be found that some characteristic peaks tend to decrease and even disappear, and six modes located at 202 cm^−1^, 279 cm^−1^, 318 cm^−1^, 416 cm^−1^, 537 cm^−1^ and 643 cm^−1^ can be found in the Bi_2_Fe_4_O_9_ powder with 15 min etching time. When increasing the etching time to 30 min, five modes can be detected, which are located at 202 cm^−1^, 279 cm^−1^, 318 cm^−1^, 416 cm^−1^, and 537 cm^−1^, respectively. As further increasing the etching time to 45 min, only two modes located at 202 cm^−1^ and 279 cm^−1^ can be observed. In conjunction with the results of XRD phase identification, it can be confimed that the phase remains unchanged.

**Figure 2:**
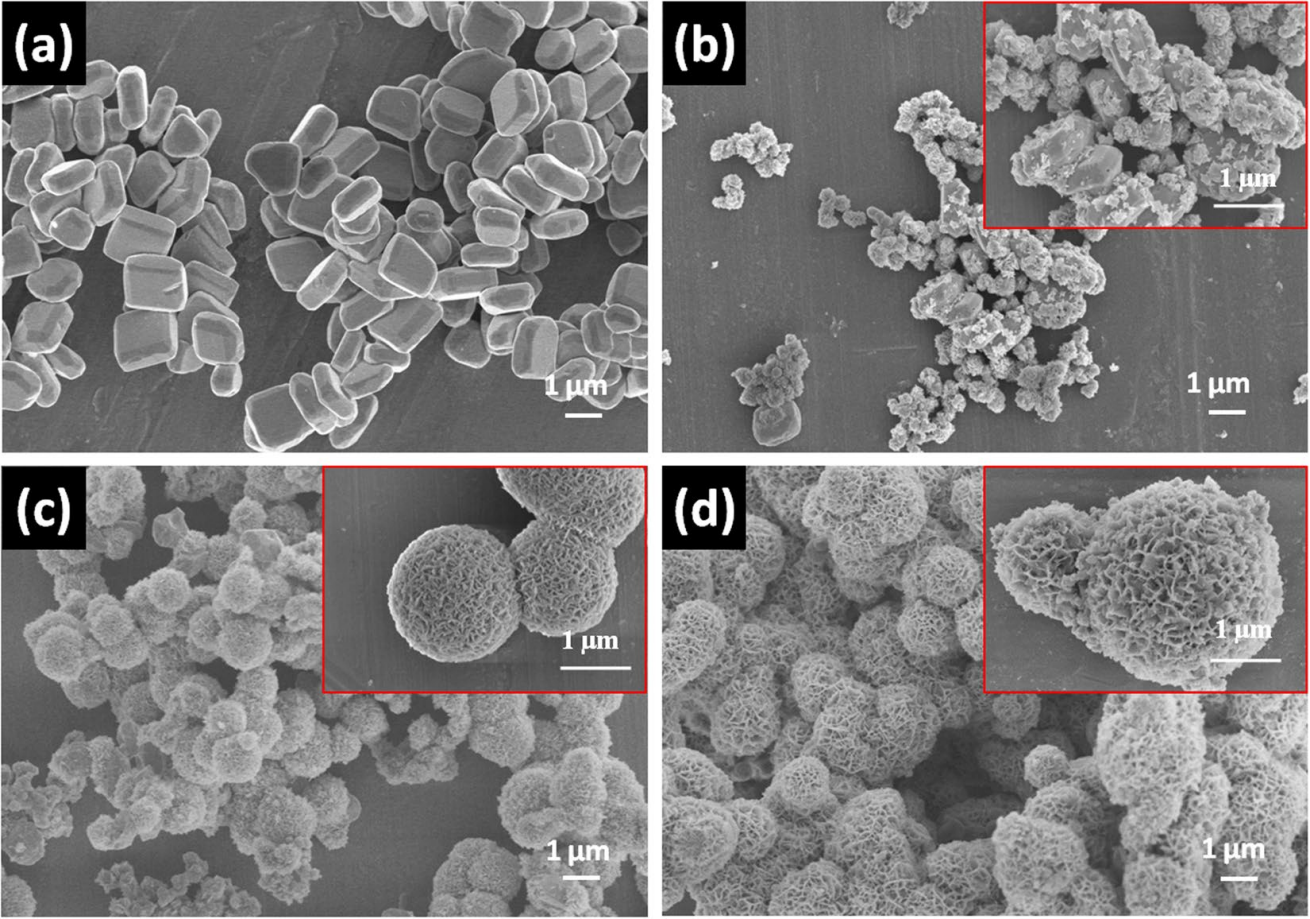
SEM images of the obtained Bi_2_Fe_4_O_9_ samples with different etching times: (**a**) 0; (**b**) 15 min; (**c**) 30 min; (**d**) 45 min.

The morphology and microstructure of the as-prepared Bi_2_Fe_4_O_9_ spheres were analyzed by the field emission scanning electron microscopy (FESEM) and transmission electron microscopy (TEM). It can be seen from Fig. 2a that the bulk Bi_2_Fe_4_O_9_ microplatelets of 1~2 μm in edge length and 300~500 nm in thickness are well dispersed with a good monodispersity, and after being etched for 15 min, the small fragments of Bi_2_Fe_4_O_9_ are produced on the surface of the bulk Bi_2_Fe_4_O_9_, and the smooth surfaces have become rough (Fig. 2b). As increasing the etching time to 30 min, the bulk Bi_2_Fe_4_O_9_ are completely etched to the small fragments and gathered together to form the flower-like nanostructure constructed with separately intersected nanosheets (Fig. 2c), which is the reason why the etched samples seem to be amorphous from the XRD and Raman spectra. As shown in Fig. 2d, it can be found that the flowerlike spheres with diameters of about 1~2 μm can be clearly observed with increasing the etching time to 45 min. Correspondingly, several higher magnification SEM images demonstrate that these flowerlike structures consist of numerous Bi_2_Fe_4_O_9_ nanoplates of 200~300 nm in edge length and 4–7 nm in thickness or so. If the reaction time may be allowed to continue, the nanoplates will become shaper and more apparent in the flower-like structure but the phase constitution of Bi_2_Fe_4_O_9_ will transform to other bismuth oxides (Bi_2_O_3_, and a mixture of Bi_2_O_3_ and Bi_2_Fe_4_O_9_) (Figure S2, Supplementary Information).

**Figure 3:**
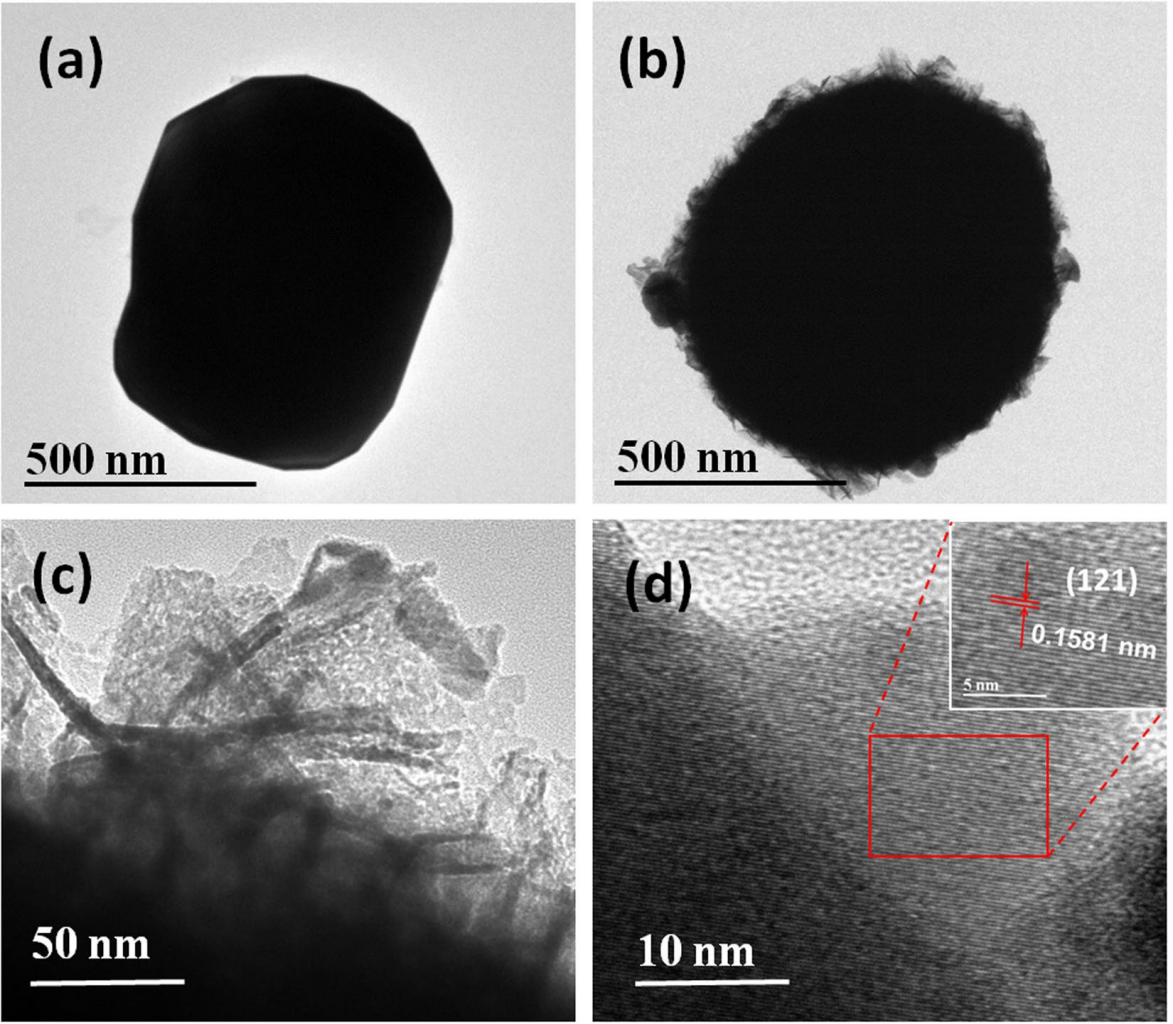
TEM images of (**a**) Bi_2_Fe_4_O_9_, (**b,c**) etched flower-like Bi_2_Fe_4_O_9_ samples and (**d**) HRTEM images of the flower-like Bi_2_Fe_4_O_9_ sample with 45 min etching.

Flower-like sphere structure was also demonstrated by TEM, as shown in Fig. 3. The typical TEM images of Bi_2_Fe_4_O_9_ spheres before and after etching are shown in Fig. 3a and b. It is shown that the evolution of morphology from microplatelets to flower-like spheres could be observed apparently after etching 45 min. Further, Fig. 3c confirms that the presence of ultrathin nanosheets in the etched Bi_2_Fe_4_O_9_ samples with a typical thickness of 4–7 nm. The structure of the as-obtained etched Bi_2_Fe_4_O_9_ spheres was investigated in more detail by high resolution transmission electron microscopy (HRTEM), as shown in Fig. 3d. The regular fringe spacing of the lattice planes is about 0.1581 nm, which is consistent with the separation of (121) plane of the orthorhombic Bi_2_Fe_4_O_9_. These observations lead to the conclusion that the bulk Bi_2_Fe_4_O_9_ crystals are exfoliated to ultrathin nanosheets by hydrazine together with methyl mercaptoacetate, which is gradually abraded from the surface of the bulk Bi_2_Fe_4_O_9_ and forms the flower-like nanostructures as the etching process continues.

**Figure 4:**
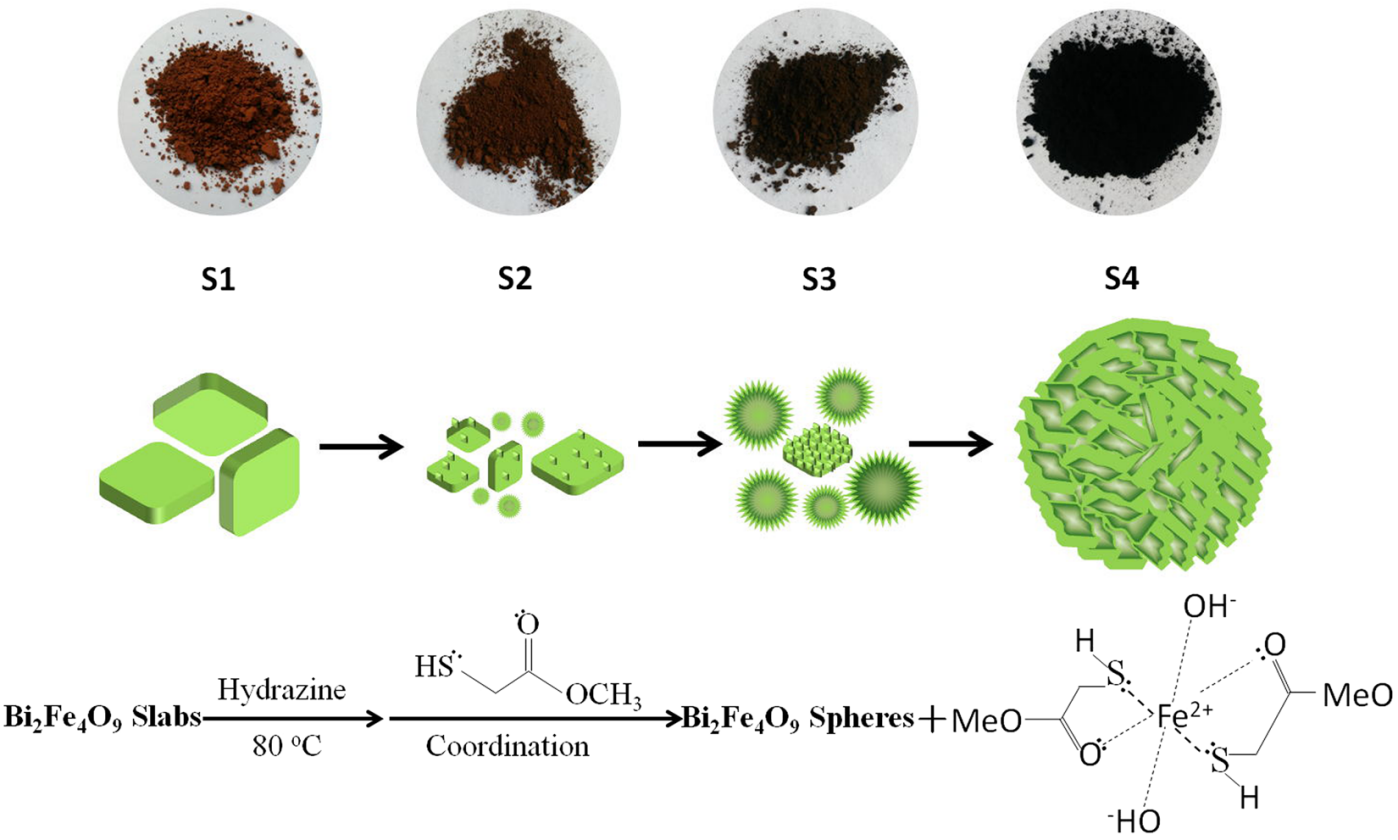
Schematic illustration of forming process of three-dimensional porous Bi_2_Fe_4_O_9_ spheres.

To analyze the changes in the Bi_2_Fe_4_O_9_ samples before and after etching, a plausible formation scheme of the Bi_2_Fe_4_O_9_ flowerlike structure spheres is illustrated in Fig. 4. Our tentative is that the evolution of ultrathin nanosheets of Bi_2_Fe_4_O_9_ may be attributed to the loss of surface Bi_2_Fe_4_O_9_ particles due to the reduction of ferric(III) to ferric(II) by hydrazine. Then, the ferric(II) is immediately coordinated with methyl mercaptoacetate in DMF, which results in the removing of Bi_2_Fe_4_O_9_. Thus, Bi_2_Fe_4_O_9_ ultrathin nanosheets are formed on surfaces of bulk Bi_2_Fe_4_O_9_. Finally, these formed nanosheets are abraded from the surfaces and gradually aggregated into flower-like spheres in virtue of the effect of surface tension. In addition, one can notice that as the amount of methyl mercaptoacetate or hydrazine changes in the reaction system, the evolution of the porous structure show a similar trend with that of the samples with different etching times (Figure S3, Supplementary Information), which confirms the above tentative.

**Figure 5:**
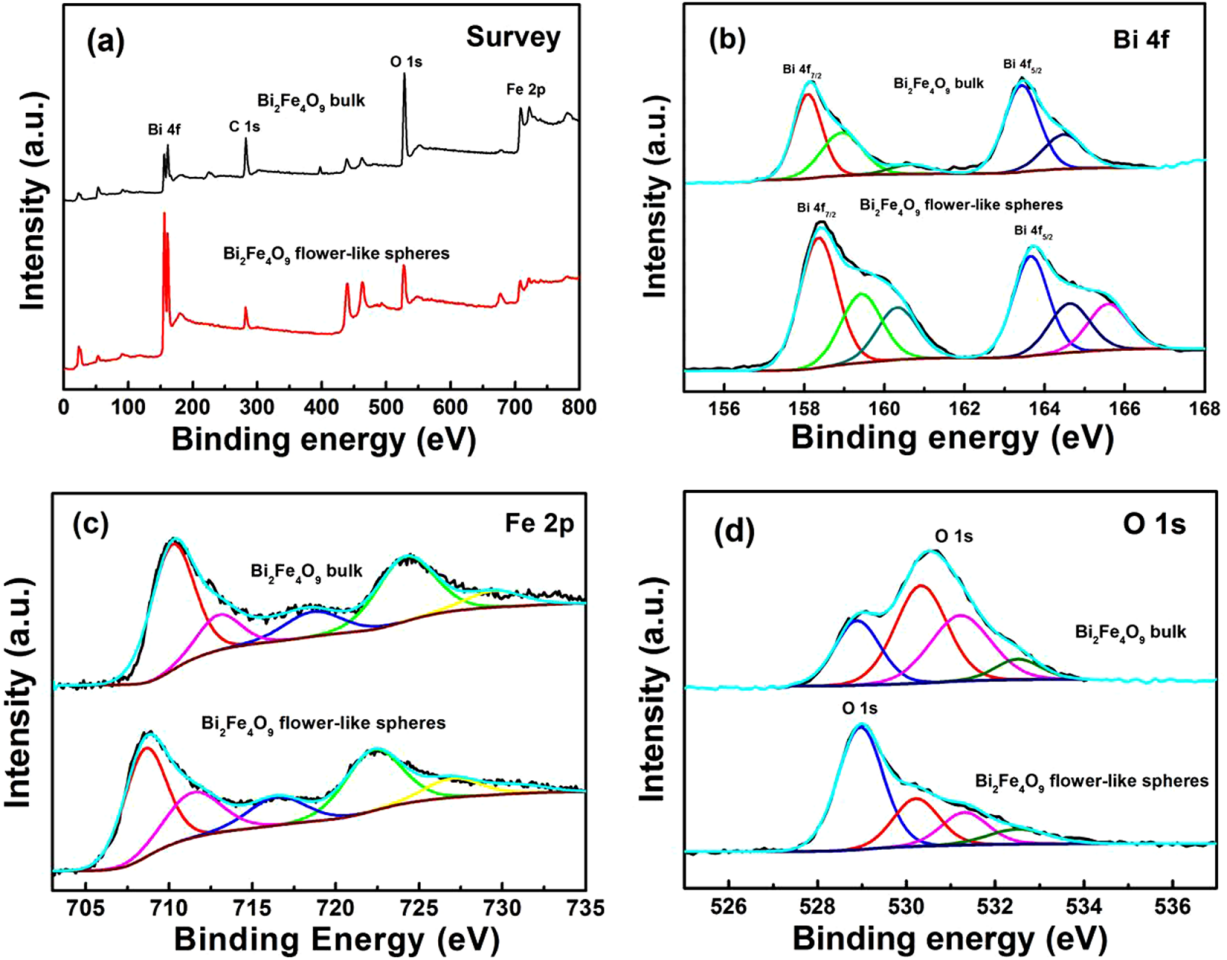
(**a**) Survey XPS spectrum of Bi_2_Fe_4_O_9_ and etched flower-like Bi_2_Fe_4_O_9_ samples; The high-resolution XPS spectra of (**b**) Bi 4 f, (**c**) Fe 2p, (**d**) O 1 s.

To investigate the chemical state of elements and the surface defects, X-ray photoelectron spectroscopy (XPS) analysis was carried out on the surface of Bi_2_Fe_4_O_9_ and etched Bi_2_Fe_4_O_9_ samples and the results are shown in Fig. 5. The obtained binding energies in XPS analysis were corrected by specimen charging which was executed by referencing the C 1 s line to 284.6 eV. It can be found that the area ratios are basically suitable for the orbital lines of Bi 4 f and Fe 2p, which is consitent with the stoichiometry of Bi_2_Fe_4_O_9_.

The survey XPS spectrum in Fig. 5a clearly reveals that both the Bi_2_Fe_4_O_9_ samples mainly consist of Bi, Fe, and O. However, the ratio of the elements in the bulk of Bi_2_Fe_4_O_9_ is different from that of the etched sample, which seems to conflict with the arguments of XRD and Raman (Table 1). It is well known that XPS analysis is only applied on the surface of powder. Presumably, a small amount of non-magnetic powder are produced and covered on the surface of Bi_2_Fe_4_O_9_ flower-like spheres, as shown in the chemical reaction schemes of Fig. 4.

**Table 1:** Content of Bi and Fe in the unetched and etched Bi_2_Fe_4_O_9_ (45 min) samples.

Sample	Bi 4 f	Fe 2p
1	7.72	3.32
2	15.83	2.44

The Bi 4 f peaks of the samples are deconvoluted into the following peaks at around 158.2 eV, 159.4 eV, 163.7 eV and 164.8 eV, respectively (Fig. 5b). By comparison, the Bi 4 f peaks of Bi_2_Fe_4_O_9_ flower-like spheres shift slightly to higher binding energy with the formation of surface bismuth defects. In addition, the appearance of two additional peaks, located at 160.4 eV and 165.8 eV, may be attributed to the signal of S 2p due to the residual sulfydryl complex on the surface of Bi_2_Fe_4_O_9_ flower-like spheres^40^. Furthermore, Bi_2_Fe_4_O_9_ is an orthorhombic structure with the coexistence of [FeO_6_] octahedra and [FeO_4_] tetrahedra^13, 31^. And the peaks at around 710.8 and 724.6 eV (Fig. 5c) are related to the binding energies of Fe 2p_3/2_ and Fe 2p_1/2_ for the ferric(III), which is perfectly consistent with the data of unetched Bi_2_Fe_4_O_9_ powders^41^. After etching, the peaks at 708.2 eV and 722.7 eV of Fe 2p corresponded with ferric(II) are detected in the etched flower-like porous Bi_2_Fe_4_O_9_ sample besides the peaks of ferric(III)^42^, suggesting the reduction of ferric(III) to ferric(II) by hydrazine. From Fig. 5d, it can be seen that the O 1 s peak is deconvoluted into four Gaussian curves at the peak positions of around 528.9 eV, 530.3 eV, 531.3 eV and 532.8 eV for the unetched Bi_2_Fe_4_O_9_ sample, which are respectively assigned to oxygen vacancies, surface lattice oxygen, ordered lattice oxygen ions^43–45^ and absorbed H_2_O or surface carbonate^46–48^. When reacted with hydrazine and methyl mercaptoacetate, the peak located at 531.3 eV and related to lattice oxygen ions is strengthened in the etched sample, further confirming that ferric(III) is reduced to ferric(II), coordinated with methyl mercaptoacetate, and dissolved in DMF and thereby the lattice oxygen vacancies are formed. The other peaks at the same positions have almost no significant change apart from weakening of peak intensity, which may be ascribed to the low crystalline of Bi_2_Fe_4_O_9_ flower-like spheres. From the above results, it is concluded that only ferric(III) ions in the Bi_2_Fe_4_O_9_ are reduced to ferric(II) and coordinated with methyl mercaptoacetate, and bismuth(III) ions is not affected.

In order to verify the above inference, the supernatant after the etching process was studied. We found that some black powders were produced in addition to the etched samples. The XPS result of the black powders (Figure S4, Supplementary Information) shows that the Fe peaks are ferric(II) and S peaks are attributed to the H-S bond and the Fe-S bond^49, 50^, indicating that ferric(III) was indeed reduced to ferric(II), coordinated with sufhydryl to form a ferric(II) complex and dissolved in dimethyl formamide (DMF). In addition, the XRD pattern of the black powders (Figure S5, Supplementary Information) shows that the complex is amorphous. The above observations draw a conclusion that the Bi_2_Fe_4_O_9_ flower-like spheres is produced by the synergistic effect of hydrazine and methyl mercaptoacetate, peeling off the surface of Bi_2_Fe_4_O_9_ to achieve numerous nanoplates.

It has been reported that larger surface area endows higher photocatalytic activity for the increased reactive sites and the promoted electron-hole separation efficiency^51–53^. With increasing the etching time, the specific surface area of Bi_2_Fe_4_O_9_ particles become larger, which may be attributed to the changing of morphology. The nitrogen adsorption-desorption isotherms of the Bi_2_Fe_4_O_9_ samples are shown (Figure S6, Supplementary Information). It can be discovered that the etched Bi_2_Fe_4_O_9_ samples exhibit apparent hysteresis loops, which are powerful evidences of high porosities. According to Brunauer-Deming-Deming-Teller classification, the etched Bi_2_Fe_4_O_9_ samples are classified as Type H3, which are caused by the heterogeneous slit-like pores. A table shows the variation of surface area and pore volume which was calculated using the Brunauer-Emmet-Teller (BET) equation (Table S1, Supplementary Information). The surface area enlarges gradually with increasing the etching time, and reaches 41.04 m^2^/g after 45 min, which is significantly greater than that of bulk Bi_2_Fe_4_O_9_ (0.84 m^2^/g). It indicates that the formation of porous structure through etching process indeed could elevate the surface area of the bulk Bi_2_Fe_4_O_9_ effectively, achieving a marked improvement of Bi_2_Fe_4_O_9_ photocatalytic performance. Furthermore, the increased surface area would probably lead to band gap narrowing, enhancing its photocatalytic performance.

**Figure 6:**
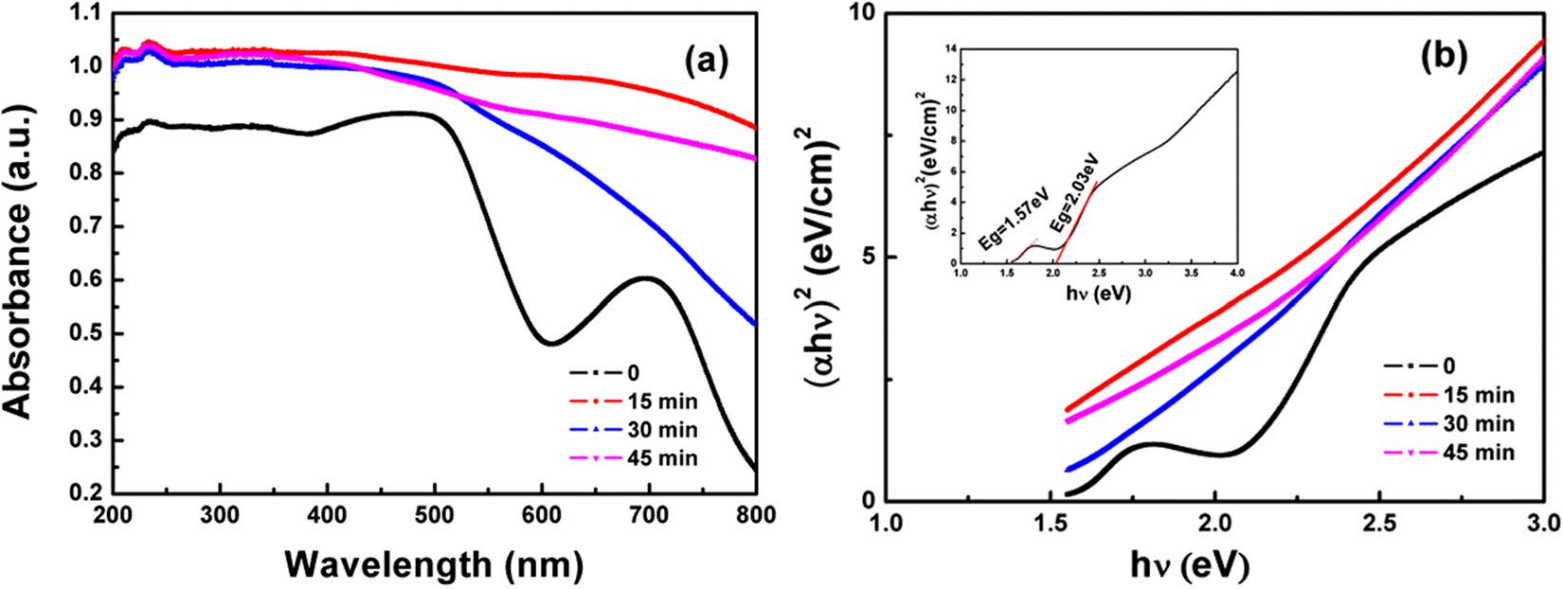
(**a**) UV-visible absorption spectra and (**b**) Degradation kinetics linear simulation curve in the presence of Bi_2_Fe_4_O_9_ with different etching times.

The diffuse reflection spectrum (DRS) of the as-prepared Bi_2_Fe_4_O_9_ samples are demonstrated to ensure the absorbance of light in the Bi_2_Fe_4_O_9_ particles before and after etching, as shown in Fig. 6a. It is shown that all the Bi_2_Fe_4_O_9_ samples can respond in the UV-vis light area. For the bulk Bi_2_Fe_4_O_9_, there are two distinct absorption edges at the wavelength of 610 nm and 850 nm in the visible region, which is consistent with the relevant literatures^13, 26^, however the absorption peaks disappear after etching. Theoretically, the movement of the absorption edge towards the lower energy visible light area may due to the narrowed bandgap width and improve visible light absorption performance. According to the results of XRD and Raman, it can be seen that the crystal structure of Bi_2_Fe_4_O_9_ are not affected by etching. Therefore, the speculation of bandgap narrowing is false. The etching process indeed leads to the enhancement of BET surface and thereby forms a porous structure to improve the light absorbance in the wavelength range from 200–800 nm compared with the unetched sample. The original absorption peak has been obscured completely by the light absorbance. It can be clearly observed that the powder color shows a tendency of darkening with increasing the etching time, as shown in Fig. 1. In addition, some mid-gap states resulted by the etching-introduced defects are beneficial for electron hopping and thus contribute to the ability of photodegradation^54^. Figure 6b shows plots of the Kubelka-Munk remission function (i.e., relationship of [αhν]^2^ versus photon energy) corresponding to each spectrum. Form Fig. 6b, it can be seen that the two bandgaps of the bulk Bi_2_Fe_4_O_9_ are calculated to be 2.01 and 1.57 eV, respectively, matching well with those of the previously reported Bi_2_Fe_4_O_9_^13, 30^. After etching process, the curves of the flower-like Bi_2_Fe_4_O_9_ samples also reveal a very strong light absorbance, implying the possibility of utilizing more light as compared with the bulk Bi_2_Fe_4_O_9_.

**Figure 7:**
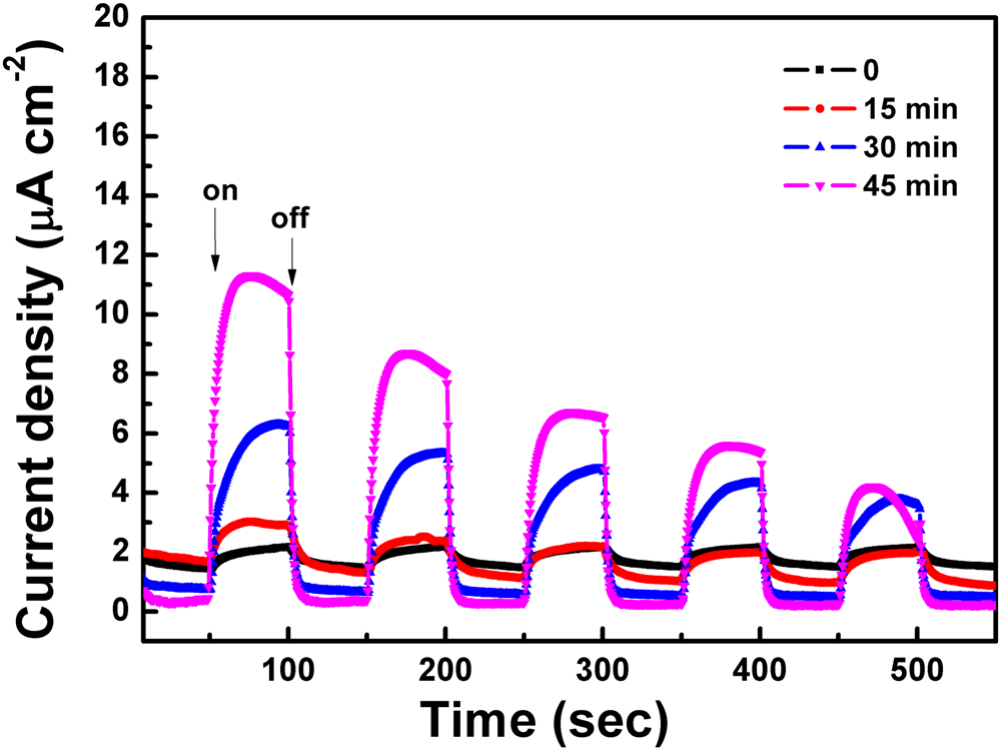
Transient photocurrent responses of the obtained Bi_2_Fe_4_O_9_ samples with different etching times.

In order to further account for the higher activity of Bi_2_Fe_4_O_9_ flower-like spheres, their photoelectrochemical response has been measured^21, 55–57^. As shown in Fig. 7, a fast and uniform photocurrent response is observed for each switch-on/off event in both photocatalysts-deposited electrodes under UV-light, and the response is entirely reversible. It also can be found that the etched Bi_2_Fe_4_O_9_ flower-like spheres exhibit much higher photoelectric current response than the unetched counterpart, which indicates that etching process endows materials higher ability of charge generate and separation. Nevertheless, there seem to be a downward trend for photocurrent densities of all the samples with increasing the test time, which may be ascribed to the weakness of Bi_2_Fe_4_O_9_ self-photoelectric translation properties.

In order to investigate the photocatalytic activity of the Bi_2_Fe_4_O_9_ samples with different morphologies, the absorption changes of MO in the presence of Bi_2_Fe_4_O_9_ with different etching times under UV-light irradiation are obtained (Figure S7, Supplementary Information). It is obvious that the as-prepared samples exhibit a clearly degradation phenomenon to MO solution under UV-light. In addition, the etched Bi_2_Fe_4_O_9_ samples exhibits a faster degradation rate than the unetched counterpart. Prior to illumination, the optical absorbance of all the samples has already reduced about 30%, which is ascribed to the adsorption of the dye molecules over Bi_2_Fe_4_O_9_, arising from the respectively specific surface area (BET area: 0.84, 12.56, 20.86, 41.04 m^2^/g). During the photodegradation process, the characteristic absorption peak at wavelength of 465 nm reduces significantly with the irradiation time. What is more, it can be observed that as increasing the etching time from 0 to 30 min, the maximum absorption peak decreases gradually from 0.51 to 0.18 after 3 h irradiation. When the etching time reaches 45 min, virtually MO in the presence of solution is degraded after 3 h illumination. However, the maximum absorption peak of the bulk Bi_2_Fe_4_O_9_ powders merely reaches 0.51 with the same irradiation time. That is to say, the porous structure may contribute to degradation of MO contaminant, which is corresponded with the previous inference.

**Fig. 8:**
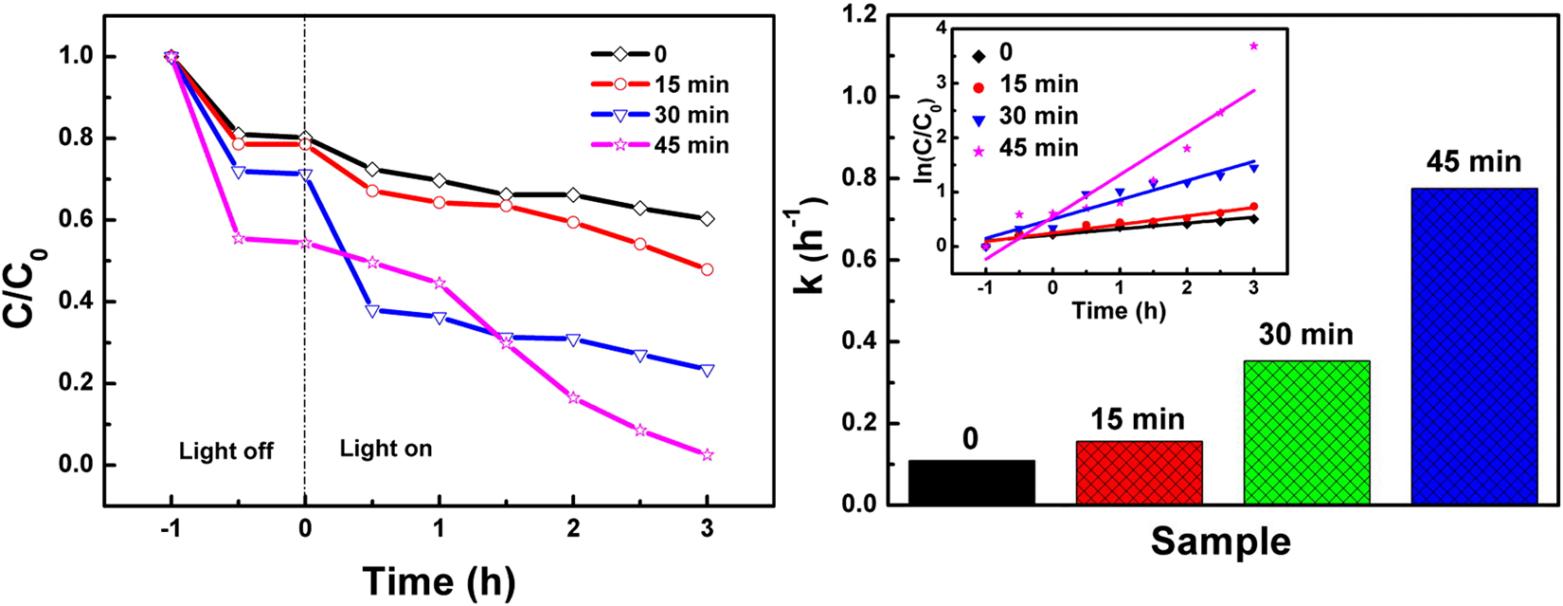
(**a**) Photodegradation efficiencies and (**b**) First-order degradation rate constant of MO in the presence of Bi_2_Fe_4_O_9_ with different etching times under UV-light illumination.

According to the data of above absorbance curve, the photodegradation efficiencies of MO in the presence of Bi_2_Fe_4_O_9_ with different etching times under UV-light illumination are shown in Fig. 8a. As is well known, the bigger the specific surface area of Bi_2_Fe_4_O_9_ is, the larger the absorbing capacity is. Figure 8a shows the degradation rate of Bi_2_Fe_4_O_9_ with different specific surface areas after 3 h UV-light irradiation. Compared with the flower-like spheres, the bulk Bi_2_Fe_4_O_9_ exhibits a much poorer effect under the same condition. Hence, it is further proved that a larger BET surface is conducive to the enhancement of photodegradation rate. Meanwhile, the photodegradation efficiencies of MO in the presence of Bi_2_Fe_4_O_9_ samples with different etching times under visible light illumination are shown (Figure S8, Supplementary Information). It cannot be found that the Bi_2_Fe_4_O_9_ samples possess evident photocatalytic activity under visible light irradiation whether by etching process or not.

The kinetics of the photoreaction can be described as being of pseudo first-order ln(C/C_0_) = kt. C_0_ and C correspond to the concentrations at t = 0 and after time “h”, respectively. Figure 8b displays the first-order degradation rate constant k(h^−1^), determined from Fig. 8a, and of all the samples, are shown the first-rank photocatalytic activity efficiency. The calculated rate constant k (h^−1^) in the presence of Bi_2_Fe_4_O_9_ with different etching time (0, 15 min, 30 min, 45 min) are 0.07, 0.15, 0.49, 0.84 h^−1^, respectively, indicating that the photocatalytic activity of the etched Bi_2_Fe_4_O_9_ samples were evidently improved by dozens of times compared with the bulk Bi_2_Fe_4_O_9_ sample.

**Figure 9:**
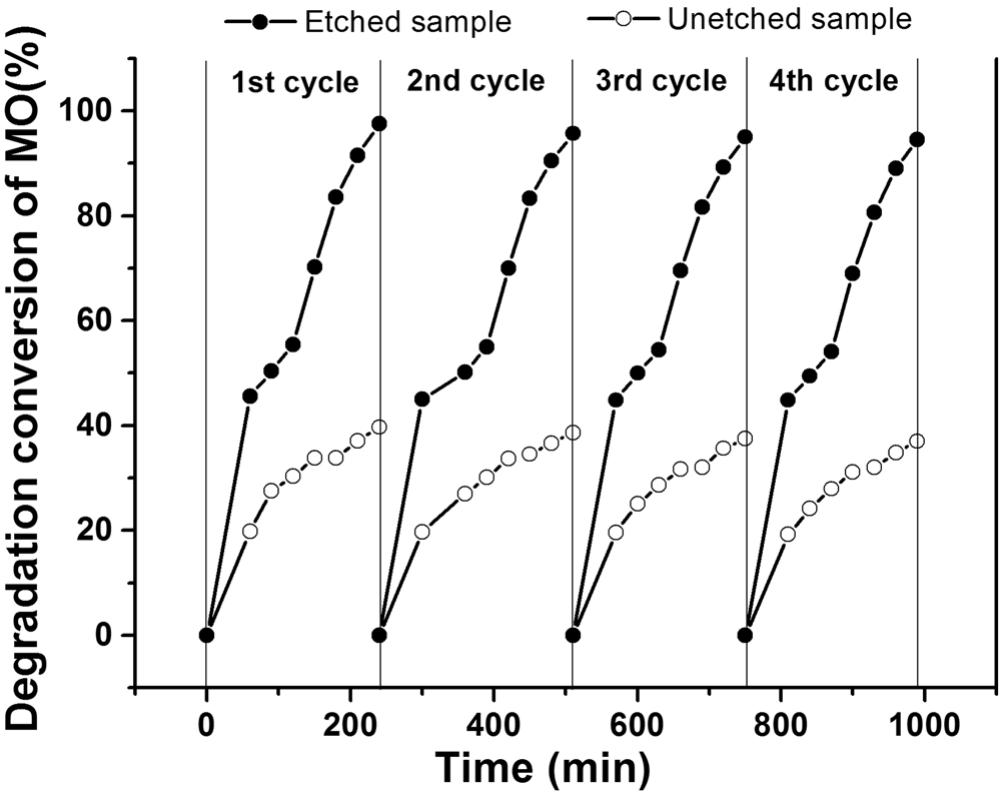
Long-term catalytic stability of the bulk Bi_2_Fe_4_O_9_ and the flower-like Bi_2_Fe_4_O_9_ spheres in repetitive experiments of MO degradation under UV light irradiation.

The stability of photocatalysts is also important for paractical application. To investigate the stability of the as-prepared photocatalysts, the repeatability experiments of MO degradation over the bulk Bi_2_Fe_4_O_9_ and the flower-like Bi_2_Fe_4_O_9_ spheres was conducted. The results are shown in Fig. 9 and no significant change is observed, indicating that the photocatalyst has an excellent stability after four recycling runs.

## Method

### Materials

Bismuth nitrate (Bi(NO_3_)_3_·5H_2_O), iron nitrate (Fe(NO_3_)_3_·9H_2_O), sodium hydroxide (NaOH), concentrated nitric acid (HNO_3_, 65%) methyl mercaptoacetate anhydrous ethanol and DMF were purchased from Sinopharm Chemical Reagent Corp (Shanghai, China) and used as received. Hydrazine (NH_2_NH_2_·H_2_O) and methyl orange were purchased from Aladdin (Shanghai, China) used as received. Anhydrous ethanol and deionized water were used in all the experiments. All the chemicals used in this study were analytical grade and were used without further purifications.

### Synthesis of pure-phase Bi_2_Fe_4_O_9_

Bi_2_Fe_4_O_9_ microplatelets were synthesized via the hydrothermal reaction. Stoichiometric Bi(NO_3_)_3_·5H_2_O (1 mmol) and Fe(NO_3_)_3_·9H_2_O (2 mmol) were dissolved in 10 mL of diluted nitric acid to form an aqueous solution under vigorously magnetic stirring at room temperature. After 10 minutes, 12 mol/L of NaOH solution, was added dropwise into the solution under vigorous stirring continuously for 30 min and a brown suspension liquid was formed. Then, the solution was transferred to a 100 mL Teflon-lined steel autoclave and maintained at 200 °C for 12 h. After being cooled to room temperature, the obtained precipitate was centrifuged and washed with absolute alcohol and water several times, and then dried 60 °C for 12 h.

### Synthesis of three-dimensional porous Bi_2_Fe_4_O_9_ spheres

Based on the Bi_2_Fe_4_O_9_ microplatelets, three-dimensional porous Bi_2_Fe_4_O_9_ spheres were synthesized by a facile etching process. Bi_2_Fe_4_O_9_ (500 mg) was dispersed ultrasonically in DMF (150 mL) in a 250 mL reagent bottle, and then a certain amount of hydrazine (6 mL) and methyl mercaptoacetate (1.5 mL) were added. After N_2_ protection for 30 min, the mixed solution reacted at 80 °C in a water-bath. The reaction time of etching process was 15 min, 30 min, 45 min for the preparation of samples. The reaction was terminated by cold ethanol and then the sediment was immediately washed by ethanol and deionized water several times respectively, followed by drying in vacuum for 12 h. For the synthesis of etching Bi_2_Fe_4_O_9_ samples with different amounts of hydrazine and methyl mercaptoactate, the reaction time was fixed at 45 min and the ratio of amounts of hydrazine and methyl mercaptoactate was also fixed at 4:1. A experiment that the amount of hydrazine and methyl mercaptoacetate was 4 mL and 1 mL respectively, has also been done.

### Characterization

The phases of the samples were analyzed by X-ray diffraction (XRD, D/max-2200, Rigaku, Japan) using Cu Kα radiation (λ = 0.15418 nm) and Raman spectra recorded at room temperature using a micro-Raman spectrometer (ALMEGA-TM, Thermo Nicolet, American) in the backscattering geomitry with a 532 nm Ar^+^ laser as an excitation source. And the morphology and characterization of the samples were observed by field emission scanning electron microscopy (FE-SEM, Quanta 250FEG, FEI, USA) and transmission electron microscope (TEM, JEM-2100, JEOL, Japan). X-ray photoelectron spectroscopy (XPS) measurements were performed by using an ultrahigh vacuum VG Scientific Corp MK-II electron spectrometer equipped with a multichannel detector. The spectra were excited using Mg Ka (1253.6 eV) radiation (operated at 200 W) of a twin anode in the constant analyzer energy mode with a pass energy of 50 eV. The Brunner-Emmet-Teller (BET, ASAP 2020, Micromeritics, USA) was used to calculate the specific surface area. The ultraviolet-visible diffuse reflectance spectrum (DRS, Cary 5000, Agilent, USA) was used in the wavelength range of 200–800 nm to study the absorption range of the samples. The UV-vis absorption spectra were measured on a UV-vis spectrophotometer (UV-2600A, Unico Instrument Corp, China).

### Measurements of photocatalytic activity

The photocatalytic behaviors of the as-prepared samples were evaluated by the degradation of MO under ultraviolet and visible light irradiation, respectively. The ultraviolet light source for catalytic reaction was a 300 W mercury lamp and the visible-light source was a 500 W xenon lamp positioned in a quartz cold trap which was in the middle of multiposition cylindrical reaction vessel. The system was cooled by wind and water at room temperature. In every run, 50 mg Bi_2_Fe_4_O_9_ was added to 50 mL MO solution (10^−5^ mol/L) in a Pyrex vessel. Before illumination, the suspensions were magnetically stirred in the dark for 40 min to ensure the establishment of an adsorption-desorption equilibrium between the photocatalyst and dye. During ultraviolet and visible light irradiation, a certain amount of mixed solution were withdrawn at regular time intervals and centrifuged to obtain the supematant which were analyzed the absorbance with a UV-vis spectrophotometer. Then, the repeatability experiments of MO degradation over the bulk Bi_2_Fe_4_O_9_ and the flower-like Bi_2_Fe_4_O_9_ spheres was measured under the same condition.

## Conclusions

In summary, three-dimensional Bi_2_Fe_4_O_9_ flower-like spheres were successfully fabricated through two steps containing a hydrothermal process and subsequently etching. The bulk Bi_2_Fe_4_O_9_ slabs are coordinated with methyl mercaptoacetate and hydrazine through a series of oxidation-reduction reaction, transforming to spheres with a porous flower-like structure. XRD patterns and Raman spectra analysis show that the Bi_2_Fe_4_O_9_ still express purity phase before and after the etching process. SEM, TEM and BET analysis indicate the variations of morphology and BET surface area. The appropriate etching time exhibit a great influence on the morphology of the Bi_2_Fe_4_O_9_ samples. The etching process indeed leads to a porous structure and thereby achieve the enhancement of BET surface of Bi_2_Fe_4_O_9_, which would be beneficial to the enhancement of photocatalytic performance owing to the prominent absorption in the ultraviolet and visible light region. Furthermore, some mid-gap states resulted by the etching-introduced defects are beneficial to electron hopping and thus contribute to the photodegradation of MO contaminant. The etching strategy applied in this study provides an effective method through changing morphology to improve various properties of multiferroic materials.

## Electronic supplementary material

A Novel Approach to Prepare Bi2Fe4O9 Flower-like Spheres with Enhanced Photocatalytic Performance
