# Supplementary material for: A novel approach to prepare Bi2Fe4O9 flower-like spheres with enhanced photocatalytic performance
Source: Sci Rep. 2017 Apr 10;7:768. doi: 10.1038/s41598-017-00831-3 (PMC5429721; doi:10.1038/s41598-017-00831-3)
Supplement: Supplementary file 1 — A Novel Approach to Prepare Bi2Fe4O9 Flower-like Spheres with Enhanced Photocatalytic Performance [file 41598_2017_831_MOESM1_ESM.doc]

**Supplementary Informations:**

A Novel Approach to Prepare Bi_2_Fe_4_O_9_ Flower-like Spheres with Enhanced Photocatalytic Performance

Haibo Yang[[1]](#footnote-2)*, Jingjing Dai, Lei Wang, Ying Lin, Fen Wang, Pan Kang

*School of Materials Science and Engineering, Shaanxi University of Science and Technology, 710021，Xi’an, PR China.

AUTHOR INFORMATION

Corresponding Author (*Haibo Yang)

E-mail: yanghaibo@sust.edu.cn.


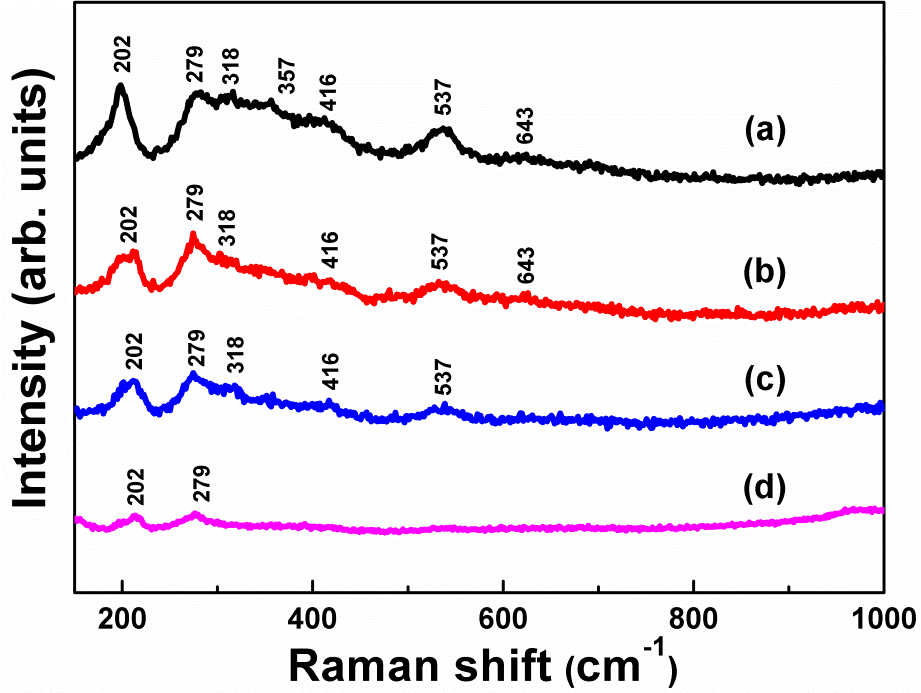


Fig. S1 Raman patterns of the obtained Bi_2_Fe_4_O_9_ samples with different etching time: (a) 0; (b) 15 min; (c) 30 min; (d) 45 min


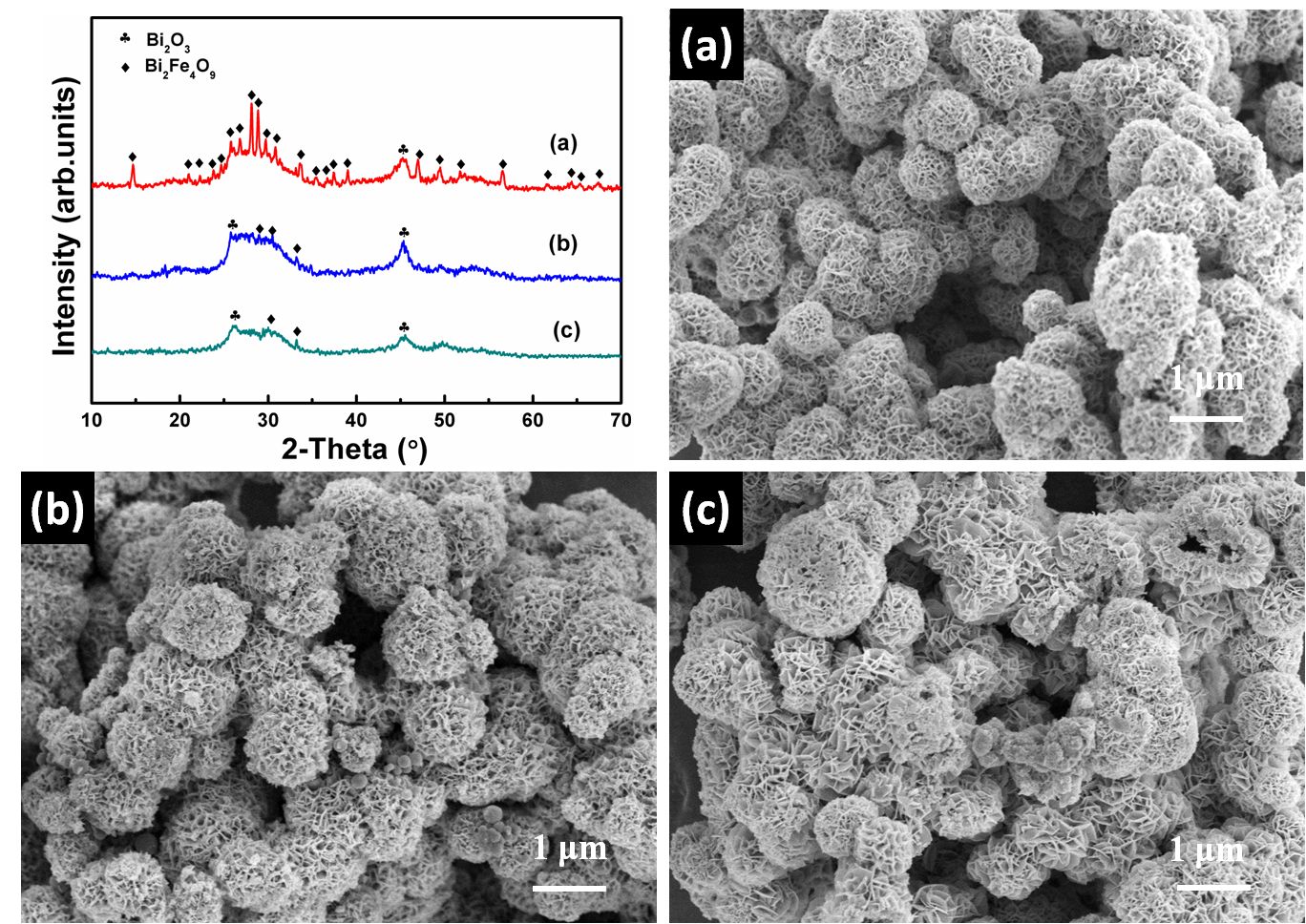


Fig. S2 XRD pattern and SEM images of the obtained Bi_2_Fe_4_O_9_ samples with longer etching times: (a) 45 min; (b) 60 min; (c) 90 min


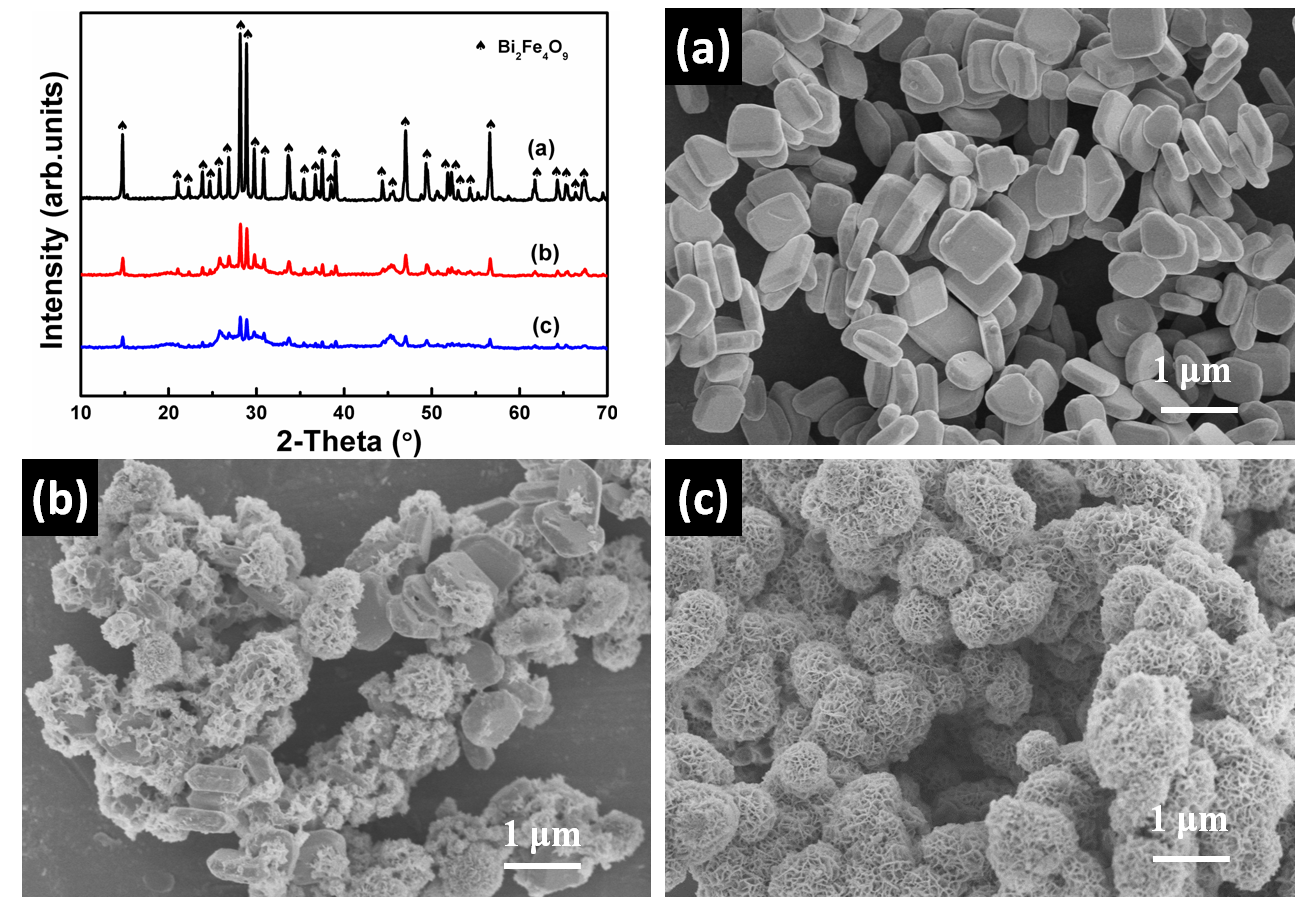


Fig. S3 XRD pattern and SEM images of etching Bi_2_Fe_4_O_9_ samples under the etching time of 45 min with different amounts of hydrazine and methyl mercaptoacetate: (a) 0; (b) 4 mL and 1 mL; (c) 6 mL and 1.5 mL


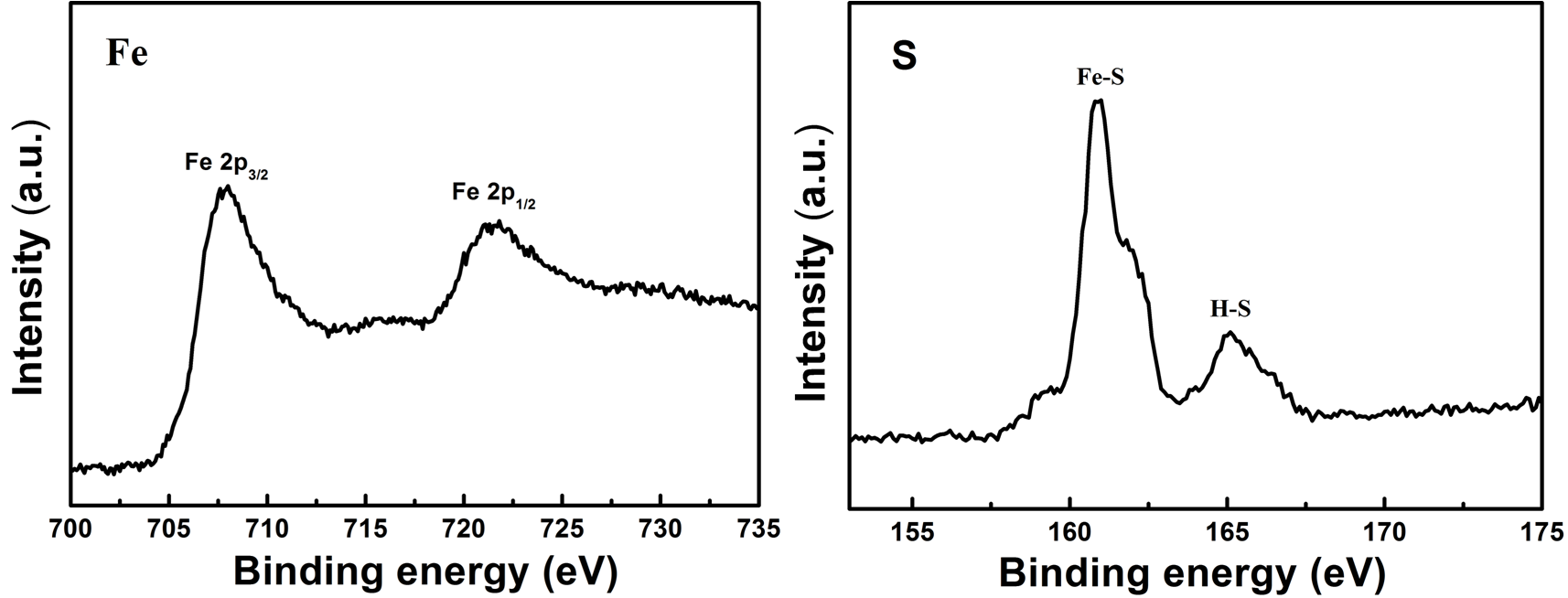


Fig. S4 Survey XPS spectrum of Fe element and S element in the black powders that were separated from supernatant


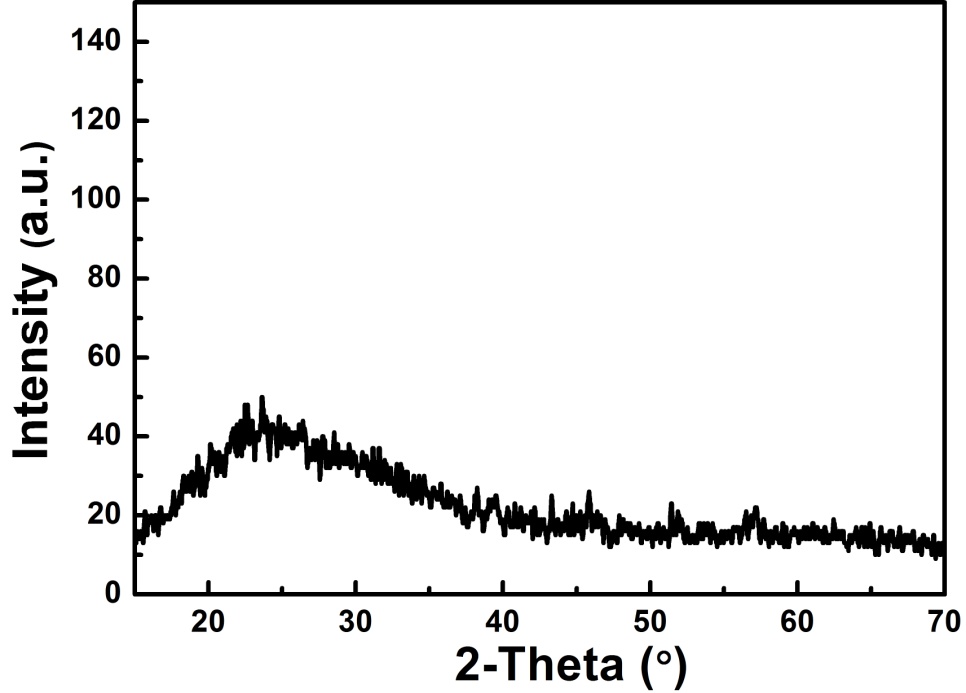


Fig. S5 XRD patterns of the black powders that were separated from supernant


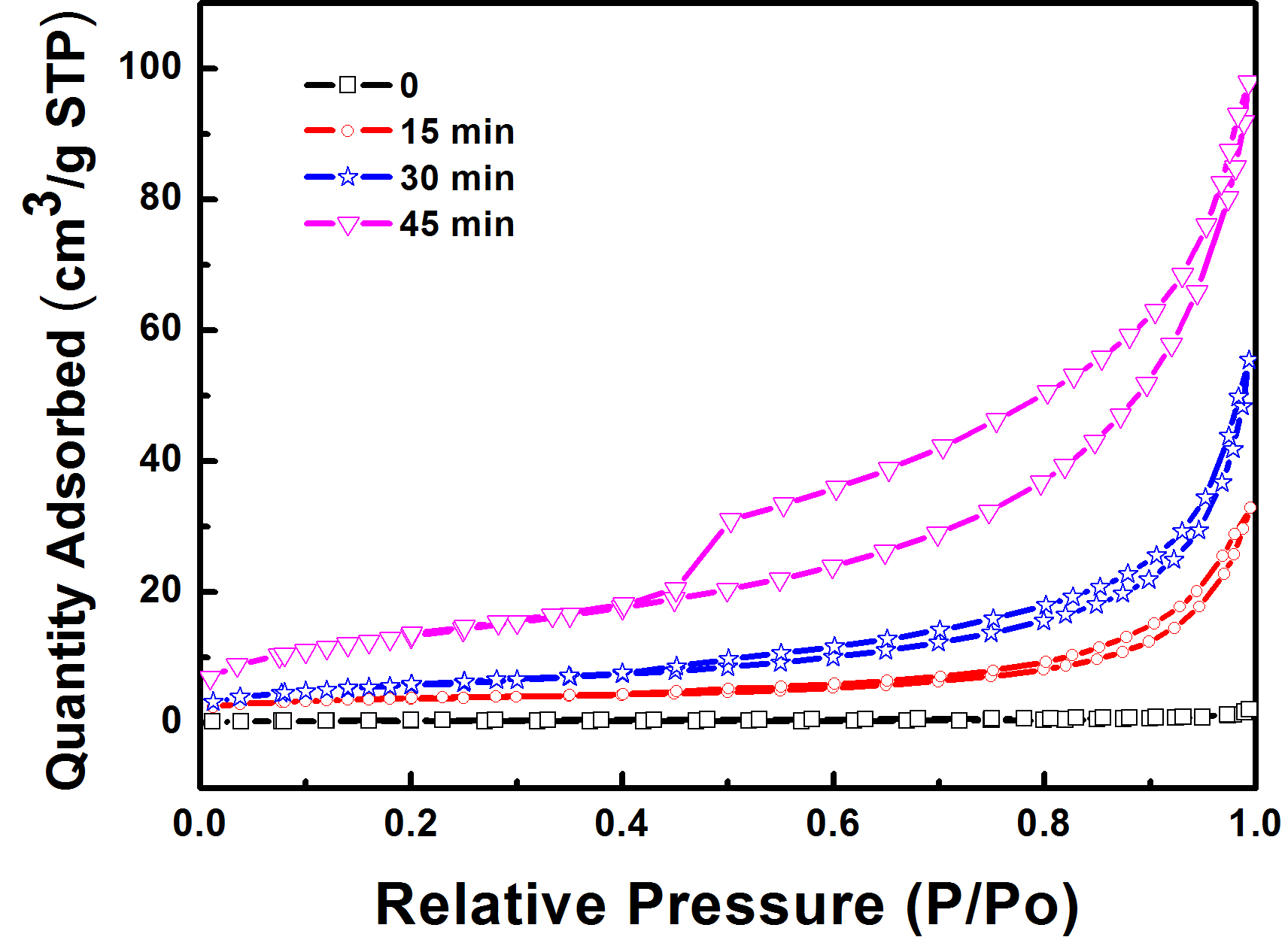


Fig. S6 N_2_ adsorption-desorption isotherm of the obtained Bi_2_Fe_4_O_9_ samples with different etching times


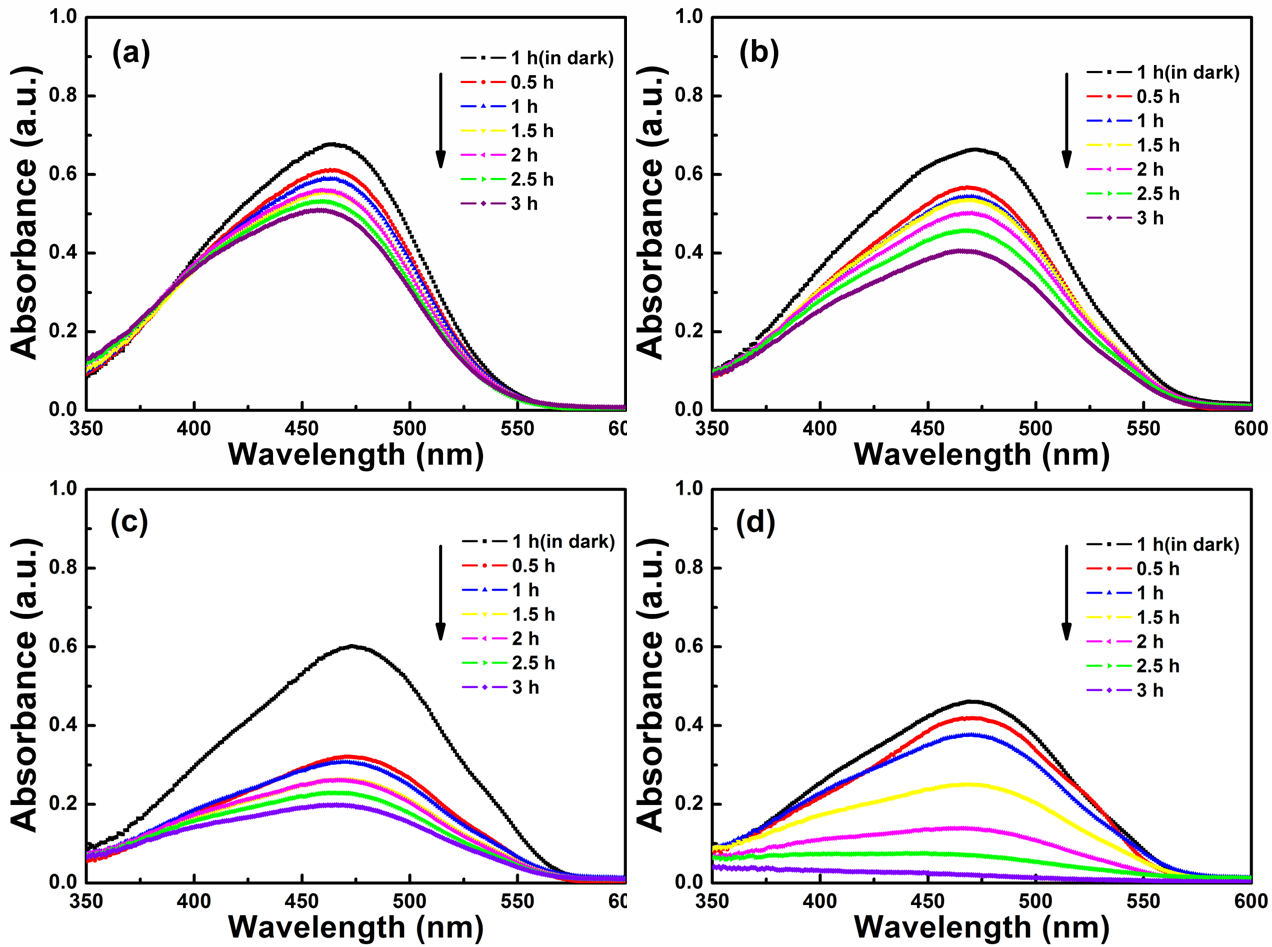


Fig. S7 Absorption change of methyl orange(MO) of MO under the UV-light over the obtained Bi_2_Fe_4_O_9_ with different etching times: (a) 0; (b) 15 min; (c) 30 min; (d) 45 min


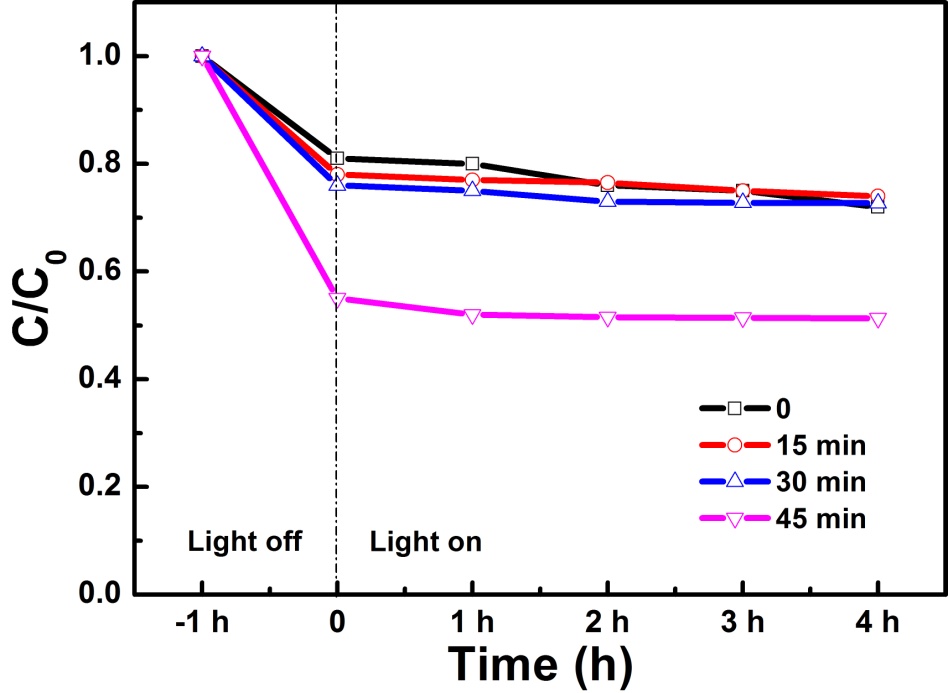


Fig. S8 Photodegradation efficiencies of MO in the presence of Bi_2_Fe_4_O_9_ with different etching times under visible light illumination

Table S1 Surface area and pore volume of the obtained Bi_2_Fe_4_O_9_ samples with different etching times: (a) 0; (b) 15 min; (c) 30 min; (d) 45 min

| **Etching time (min)** | **Crystal form** | **Morphology** | **Pore volume (cm^3^/g)** | **Specific surface area (m^2^g)** |
| --- | --- | --- | --- | --- |
| **0** | **orthorhombic** | **slabs** | **0.003** | **0.84** |
| **15** | **orthorhombic** | **slabs+fragments** | **0.050** | **12.56** |
| **30** | **orthorhombic** | **spheres** | **0.085** | **20.86** |
| **45** | **orthorhombic** | **spheres** | **0.151** | **41.04** |

1. ^^ Corresponding author. Tel: +86-29-86168688; Fax: +86-29-86168688; Email: yanghaibo@sust.edu.cn [↑](#footnote-ref-2)
